# Supplementary material for: A Guide for Using Transmission Electron Microscopy for Studying the Radiosensitizing Effects of Gold Nanoparticles In Vitro
Source: Nanomaterials (Basel). 2021 Mar 27;11(4):859. doi: 10.3390/nano11040859 (PMC8065702; doi:10.3390/nano11040859)
Supplement: Supplementary file 1 [file nanomaterials-11-00859-s001.pdf]

nanomaterials

MDPI

Supplementary Material

A Guide for Using Transmission Electron Microscopy for Studying the Radiosensitizing Effects of Gold Nanoparticles In Vitro

Ioanna Tremi [1,2], Sophia Havaki [2], Sofia Georgitsopoulou [3], Nefeli Lagopati [2], Vasilios Georgakilas [3], Vassilis G. Gorgoulis [2,4,5,6] and Alexandros G. Georgakilas [1]

1 DNA Damage Laboratory, Department of Physics, School of Applied Mathematical and Physical Sciences, National Technical University of Athens (NTUA), Zografou Campus, 15780 Athens, Greece; ioannatremi@mail.ntua.gr (I.T.); alexg@mail.ntua.gr (A.G.G.)
2 Molecular Carcinogenesis Group, Department of Histology and Embryology, School of Medicine, National and Kapodistrian University of Athens, 75 Mikras Asias Street, 11527 Athens, Greece; shavaki@med.uoa.gr (S.H.); nlagopati@med.uoa.gr (N.L.); vgorg@med.uoa.gr (V.G.G.)
3 Department of Material Science, University of Patras, 26504 Patras, Greece; up1057156@upatras.gr (S.G.); viegeorgaki@upatras.gr (V.G.)
4 Biomedical Research Foundation, Academy of Athens, 4 Soranou Ephessiou Street, 11527 Athens, Greece
5 Faculty Institute for Cancer Sciences, Manchester Academic Health Sciences Centre, University of Manchester, Manchester, MP13 9PL, UK
6 Center for New Biotechnologies and Precision Medicine, Medical School, National and Kapodistrian University of Athens, 75 Mikras Asias Street, 11527 Athens, Greece
* Correspondence: alexg@mail.ntua.gr

1 Protocols

1.1. Cell Incubation with gold nanoparticles

- Dilute gold nanoparticles in full cell growth medium in order to reach desired concentration. After cells are attached to the surface, replace old medium with fresh full growth medium containing nanoparticles. Add 3-5ml for 60mm dishes or 10ml for 100mm dishes. Cells are incubated with nanoparticles for 24h.

- After 24h, wash cells x3 times with 0.01M PBS.

- After PBS resuspend cells with fresh full growth medium. Add 5ml for 60mm dishes or 10-15ml for 100mm dishes.

1.2. Cell fixation

- Remove growth medium and wash cells x1 with 0.01M PBS.

- Fix cells for 30min with appropriate aldehyde solution. Use 2.5% glutaldehyde in 0.01M PBS for TEM observation or 3% paraformaldehyde and 0.5% glutaldehyde in 0.1M PB for immunocytochemistry purposes before TEM observation. ! NOTE All solutions must be in RT before use. ! CAUTION Fixation must be performed under a fume hood.

- After fixation, wash cells x1 for 30sec and x3 for 5min with 0.01M PBS. ! NOTE If fixative is in PB, use 0.1PB instead of PBS to wash cells.

- Resuspend cells in fresh PBS. Use 5ml for 60mm dishes or 10ml for 15mm dishes and place them at 4°C until further steps.

1.3. Embedding cells in gelatin and cutting pieces for processing

- Remove old PBS and collect cells in a 2ml eppendorf tube using a cell scraper. Scrape cells x2 while adding 1ml of 0.01M PBS before every scraping. Collect cells using a glass pasteur pipette.

- Centrifuge cells in a microcentrifuge for 5min at 800g or 1000g (depending on the cell line) so that they create a cell pellet. Remove supernatant PBS and add 1ml of 4% fresh gelatin aqueous solution. ! NOTE Gelatin solution must be warmed up to be watery.

- Centrifuge cells again for 5min at same speed.

- Place eppendorf tube in ice until gelatin becomes solidified. Place also glass vials with PBS on ice (use as many as your experimental conditions).

- Cut tube using a one-side razor blade and place gelatin cell pellet on a glass petri dish under ice. Under a stereoscope, short out the cell pellet from the gelatin and cut small cell pieces (cubic shape) of ~1mm thickness. Place pieces in glass vials containing cold PBS and close them tight. Place vials at 4°C until further steps. ! NOTE Cutting must be done quickly so that gelatin doesn't liquefy due to heat.

1.4. Embedding cells in epoxy resins

Glass vials and caps used for the following chemical procedures must endure fixation, dehydration, and infiltration procedures. All procedures are performed in a fume hood.

- <u>Second fixation</u>: Remove PBS and add 1% aqueous solution of OsO_4 for 1h at 4°C.

- <u>Dehydration</u>: All dehydration steps are performed sequentially using ethyl alcohol solutions (ethanol) of gradually increasing concentration, as described below:

  1. 25% ethanol, for 7 min at 4°C
  2. 50% ethanol, for 7 min at 4°C
  3. 70% ethanol, for 7 min at 4°C
  4. 95% ethanol, for 7 min at 4°C
  5. 100% ethanol, 2×10 min in RT

- <u>Infiltration</u>: All steps are carried out in RT. Before infiltration of specimens with epoxy resins, they are incubated with propylene oxide (P.O.) to facilitate the penetration of epoxy resins into them. Subsequently, specimens are infiltrated gradually with a mixture of epoxy resin/propylene oxide solutions of increasing concentration regarding epoxy resins, as described below. During infiltration, vials with specimens are placed on a rotator.

  1. P.O. 100%, 3×5 min
  2. Epoxy resins/P.O. = 1:3 for 30 min
  3. Epoxy resins/P.O. = 1:2 for 30 min
  4. Epoxy resins/P.O. = 1:1 overnight until P.O is evaporated

- <u>Embedding and Polymerization:</u>

  1. Replace old resin with 100% fresh epoxy resin and leave for 1h on a rotator.

2. Place cell pieces with an anatomy needle in flat embedding molds filled with fresh epoxy resin (1 piece in each well of the mold). Orient pieces appropriately under a stereoscope. Place the mold in an oven at 60°C for polymerization for 24h.

3. After polymerization remove the epoxy resin blocks from the mold and cut sections using an ultramicrotome.

1.5. Embedding cells in Lowicryl HM20 resin for PLT

This protocol is performed in the LEICA EM AFS instrument. Chemically fixed cell pieces are placed inside the AFS chamber in capsules that have a mesh at the bottom (mesh capsules). This PLT protocol is based on the Robertson et al protocol [1], with slight modifications. All solutions must be precooled at the corresponding temperatures before use.

- Dehydration: Steps are performed using increasing concentration of 100% anhydrous ethyl alcohol (ethanol), while progressively lowering temperature (rate: 5°C/h), as described below:

1. 30% ethanol, for 30 min at 0°C
2. 50% ethanol, for 30 min at -10°C
3. 70% ethanol, for 30 min at -25°C
4. 100% ethanol, for 30 min at -40°C
5. 100% ethanol, for 30 min at -50°C

- Infiltration: Infiltration takes place gradually by increasing concentration of acrylic resin in 100% anhydrous ethanol at -50°C. Steps are shown below:

1. Lowicryl HM20 resin/ethanol : 25%/75%, for 60 min at -50°C
2. Lowicryl HM20 resin/ethanol : 50%/50%, for 60 min at -50°C
3. Lowicryl HM20 resin/ethanol : 75%/25%, for 60 min at -50°C
4. 100% Lowicryl HM20 resin, for 60 min at -50°C
5. 100% Lowicryl HM20 resin, overnight (20 - 24h) at -50°C

- Embedding and Polymerization:

1. After infiltration replace resin with fresh 100% Lowicryl HM20 resin and incubate for 1h.
2. Place Leica capsules (beam capsules) inside the appropriate metallic containers and fill it up with Lowicryl HM20 resin.
3. Place gelatin capsules inside another metallic container and fill them up again with Lowicryl HM20 resin.
4. Transfer cell pieces from mesh capsules to Leica capsules. Each capsule corresponds to one experimental condition.
5. Transfer the Leica capsules inside the gelatin capsule container with the help of a metallic "spider" that holds simultaneously all the Leica capsules.
6. Finally transfer the entire device (containing both capsules) to a special support container filled with 15ml of 100% anhydrous ethanol.
7. Fit UV lamp on the AFS processing chamber for polymerization. Polymerize blocks first for 48h at -50°C and second for 24h at 0°C (rate: 5°C/h)).

1.6. Immunogold labelling for single or double immunolocalization

All procedures take place at RT, except from the primary antibody incubation step that is carried out at 4°C. Before use, all solutions must be filtered using syringe with a 0.22μm filter. The incubation at each step of the protocol is done by floating the grids on drops of the incubation solution, so that the side of the grids with the mounted sections comes into contact with the solution. All drops are placed in Terasaki well plates (15μl/well) with lids. Lids ensure a dust-free and moisturizing environment. Grids are transferred each time using an antimagnetic fine tip tweezer (no5 or no7).

- Incubate grids with 0.05M Tris/HCL buffer, pH 7.4
    1. 2x2min
    2. 2x1min
- Incubate grids with 0.1M glycine in 0.05M Tris/HCL buffer, pH 7.4 for 30min at RT.
- Wash grids with 0.05M Tris/HCL buffer, pH 7.4, 4x1min.
- Incubate grids with blocking solution for 30min at RT.
- Drain grids using a lens tissue, before 1ry Ab incubation step.
- Incubate grids with 1ry Ab overnight at 4°C. 1ry Ab is diluted in blocking solution at the appropriate concentration. ! NOTE 1ry Ab is diluted in blocking solution without NGS.

After 1ry Ab, Terasaki plates are placed on a magnetic stirrer for washing, in very low speed.

- Wash grids with Tris buffer I, as follows
    1. 2x2min
    2. 2x3min
    3. 5x1min
- Wash grids with Tris buffer II, 3x1min.
- Wash grids with Tris buffer III, 1x5min
- Drain grids using a lens tissue before 2ry Ab incubation step.
- Incubate grids with 2ry Ab (gold-conjugated) for 1h at RT. 2ry Ab is diluted in Tris buffer III, at the appropriate concentration. ! NOTE 2ry Ab must be centrifuged before dilution for 20min at 450g.
- After 2ry Ab, wash grids as follows
    1. Tris buffer II, 3x1min
    2. Tris buffer I, 5x1min
    3. dH_2O, 5x1min
- Drain grids very well and store them in a grid box before staining with uranyl acetate and lead citrate.

1.7 Silver-enhancement technique on thin sections

This protocol uses the HQ Silver enhancement kit from Nanoprobes. Use solutions according to the manufacturer's instructions.

- Kit is stored at -20°C and consists of 15ml initiator, 15 ml moderator and 15 ml activator. ! NOTE Leave solution in RT for 2 hours before use.
- Form reagent by combining equal volumes of the moderator, initiator, and then the activator solution. ! NOTE The developing solution is very unstable and must be protected from light.
- On a glass petri dish place clean parafilm and place some drops of filtered dH_2O. Place grids onto drop (sections facing down)) and incubate x2 for 1min.
- Drain grids on a lens tissue before the next step.
- On a piece of clean parafilm again place drops of silver reagent and place grids onto drops for as long as it takes to acquire the optimum size of particle. 6-8min incubation time gives approximately a 20nm single particle size. ! NOTE The samples should also be protected from light during silver-enhancement procedure.
- Wash grids with dH2O x2 for 5min.
- Drain grids and store until further use.

References

1. Robertson, D., et al., *An appraisal of low-temperature embedding by progressive lowering of temperature into Lowicryl HM20 for immunocytochemical studies.* J Microsc, 1992. **168**(Pt 1): p. 85-100.
